# Foodborne Transmission of Nipah Virus in Syrian Hamsters

**DOI:** 10.1371/journal.ppat.1004001

**Published:** 2014-03-13

**Authors:** Emmie de Wit, Joseph Prescott, Darryl Falzarano, Trenton Bushmaker, Dana Scott, Heinz Feldmann, Vincent J. Munster

**Affiliations:** 1 Laboratory of Virology, National Institute of Allergy and Infectious Diseases, National Institutes of Health, Hamilton, Montana, United States of America; 2 Rocky Mountain Veterinary Branch, National Institute of Allergy and Infectious Diseases, National Institutes of Health, Hamilton, Montana, United States of America; 3 Department of Medical Microbiology, University of Manitoba, Winnipeg, Manitoba, Canada; Icahn School of Medicine at Mount Sinai, New York, New York, United States of America

## Abstract

Since 2001, outbreaks of Nipah virus have occurred almost every year in Bangladesh with high case-fatality rates. Epidemiological data suggest that in Bangladesh, Nipah virus is transmitted from the natural reservoir, fruit bats, to humans via consumption of date palm sap contaminated by bats, with subsequent human-to-human transmission. To experimentally investigate this epidemiological association between drinking of date palm sap and human cases of Nipah virus infection, we determined the viability of Nipah virus (strain Bangladesh/200401066) in artificial palm sap. At 22°C virus titers remained stable for at least 7 days, thus potentially allowing food-borne transmission. Next, we modeled food-borne Nipah virus infection by supplying Syrian hamsters with artificial palm sap containing Nipah virus. Drinking of 5×10^8^ TCID_50_ of Nipah virus resulted in neurological disease in 5 out of 8 hamsters, indicating that food-borne transmission of Nipah virus can indeed occur. In comparison, intranasal (i.n.) inoculation with the same dose of Nipah virus resulted in lethal respiratory disease in all animals. In animals infected with Nipah virus via drinking, virus was detected in respiratory tissues rather than in the intestinal tract. Using fluorescently labeled Nipah virus particles, we showed that during drinking, a substantial amount of virus is deposited in the lungs, explaining the replication of Nipah virus in the respiratory tract of these hamsters. Besides the ability of Nipah virus to infect hamsters via the drinking route, Syrian hamsters infected via that route transmitted the virus through direct contact with naïve hamsters in 2 out of 24 transmission pairs. Although these findings do not directly prove that date palm sap contaminated with Nipah virus by bats is the origin of Nipah virus outbreaks in Bangladesh, they provide the first experimental support for this hypothesis. Understanding the Nipah virus transmission cycle is essential for preventing and mitigating future outbreaks.

## Author Summary

In Bangladesh, outbreaks of Nipah virus occur almost every year, resulting in respiratory and neurological disease with high case-fatality rates. Based on epidemiological data Nipah virus is thought to be transmitted from fruit bats to humans via drinking of date palm sap contaminated by bats that drink from the sap stream or collection vessel during collection. Additionally, human-to-human transmission has been shown to occur. Here, we experimentally modeled the proposed transmission cycle of Nipah virus in Bangladesh in Syrian hamsters. Hamsters that drank artificial palm sap containing high doses of Nipah virus became infected with the virus and developed neurological signs of disease. Virus replication occurred mainly in the respiratory rather than the intestinal tract. Most importantly, hamsters infected with Nipah virus through drinking of contaminated palm sap could transmit the virus to uninfected cage mates. As treatments for Nipah virus are currently unavailable and medical interventions are difficult to implement in rural outbreak areas, our best hope to prevent or intervene in future outbreaks of Nipah virus lies in the potential to block transmission from bats to humans and from human to human. Understanding how Nipah virus is transmitted is essential to achieve this.

## Introduction

Nipah virus first emerged in 1998 during a large outbreak of encephalitis and respiratory disease in Malaysia and Singapore, causing 276 cases of encephalitis with 106 fatalities [1]. Since 2001, outbreaks of Nipah virus have occurred almost every year in Bangladesh with a strikingly high case-fatality rate of up to 90% [2], with 24 cases of Nipah virus occurring to date in 2013 [3]. The recurrent outbreaks of Nipah virus in Bangladesh have been epidemiologically associated with the consumption of date palm sap, which has led to the hypothesis that Nipah virus zoonosis is a result of drinking date palm sap contaminated by infected fruit bats [4], [5]. In Bangladesh, date palm sap is harvested at nighttime from October to March [6], which overlaps with the occurrence of Nipah virus outbreaks. Although to our knowledge Nipah virus has so far not been detected in date palm sap, human observation and analysis by infrared camera has shown that bats frequently drink from date palm sap during collection [7], [8]. Since bats can shed Nipah virus in their urine and saliva [9]–[12], it is thought that bats contaminate the date palm sap while drinking from the sap stream or date palm sap collection vessel.

In addition to the initial zoonotic transmission, subsequent human-to-human transmission also plays an important role in the epidemiology of Nipah virus outbreaks in Bangladesh. It was estimated for the outbreaks in Bangladesh between 2001 and 2007 that approximately 50% of cases was the result of human-to-human transmission [13].

Thus far, the epidemiological association between drinking date palm sap and Nipah virus infection has not been confirmed experimentally. Therefore, we set out to assess the ability of date palm sap to function as a vehicle for zoonotic transmission of Nipah virus using a well-established small animal model for Nipah virus pathogenesis and transmission, the Syrian hamster [14]–[16]. We showed that, upon drinking of artificial palm sap containing high doses of Nipah virus, hamsters became infected and developed neurological signs of disease. Moreover, hamsters infected through the drinking route transmitted Nipah virus to naïve hamsters via direct contact.

## Results

### Stability of Nipah virus in artificial palm sap

**Figure 1:**
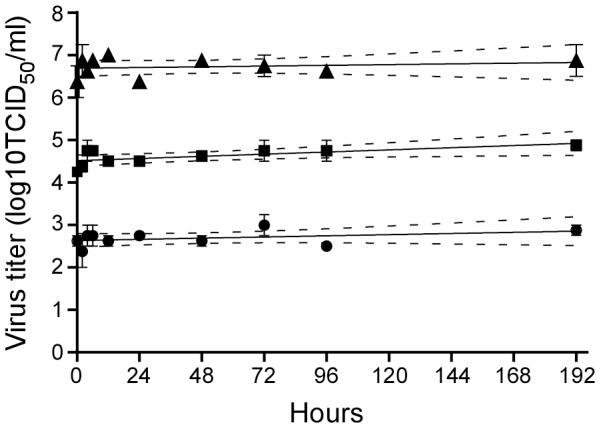
Stability of Nipah virus in artificial palm sap at 22°C. Nipah virus was diluted to 10^7^ (black triangles), 10^5^ (black squares) and 10^3^ (black circles) TCID_50_/ml in artificial palm sap and left at 22°C for 8 days. Samples were taken at the indicated time points and virus titer in those samples was determined by titration on Vero C1008 cells. The stability data were analyzed using the linear regression model in the GraphPad prism 6 software package. The regression line (solid) is plotted together with the 95% confidence interval (dotted line).

The composition of palm sap was derived from a published report [17] and artificial palm sap was produced in the laboratory consisting of 13% sucrose and 0.21% BSA in water, pH 7.0. The stability of three different doses (10^3^, 10^5^ and 10^7^ TCID_50_/ml) of Nipah virus (strain Bangladesh/200401066) in artificial palm sap was determined at 22°C. Of note, between October and March (the date palm sap harvesting season) the average temperature in Bangladesh fluctuates between 20°C and 28°C. No significant difference was detected between the slopes of the three lines (slope ± standard error: 0.001140±0.001026 for the 10^3^ TCID_50_, 0.002115±0.0008557 for the 10^5^ TCID_50_ and 0.0007124±0.001272 for the 10^7^ TCID_50_). In addition, the slopes of the three groups were also not significantly different from zero (p = 0.2812 for the 10^3^ TCID_50_, p = 0.0950 for the 10^5^ TCID_50_, p = 0.5823 for the 10^7^ TCID_50_). Thus, no significant reduction in Nipah virus infectious titer was observed at any of the dilutions for at least 8 days at 22°C (Fig. 1). At 28°C, Nipah virus titers decreased only 5–10 fold during the eight day incubation period (Fig. S1A), thus indicating preservation of Nipah virus viability in date palm sap at both temperatures.

Since heating of date palm sap before consumption has been suggested as a means of inactivating Nipah virus and thereby preventing Nipah virus infection, we tested the stability of Nipah virus in artificial palm sap at 70°C and 100°C. At both temperatures, virus titers decreased about 4 log in the first 5 minutes (Fig. S1B). However, incubation of Nipah virus in artificial palm sap at 70°C for 1 hour did not inactivate all infectious virus. In contrast, incubation at 100°C for more than 15 minutes completely inactivated Nipah virus.

### Esophageal inoculation of Syrian hamsters

**Figure 2:**
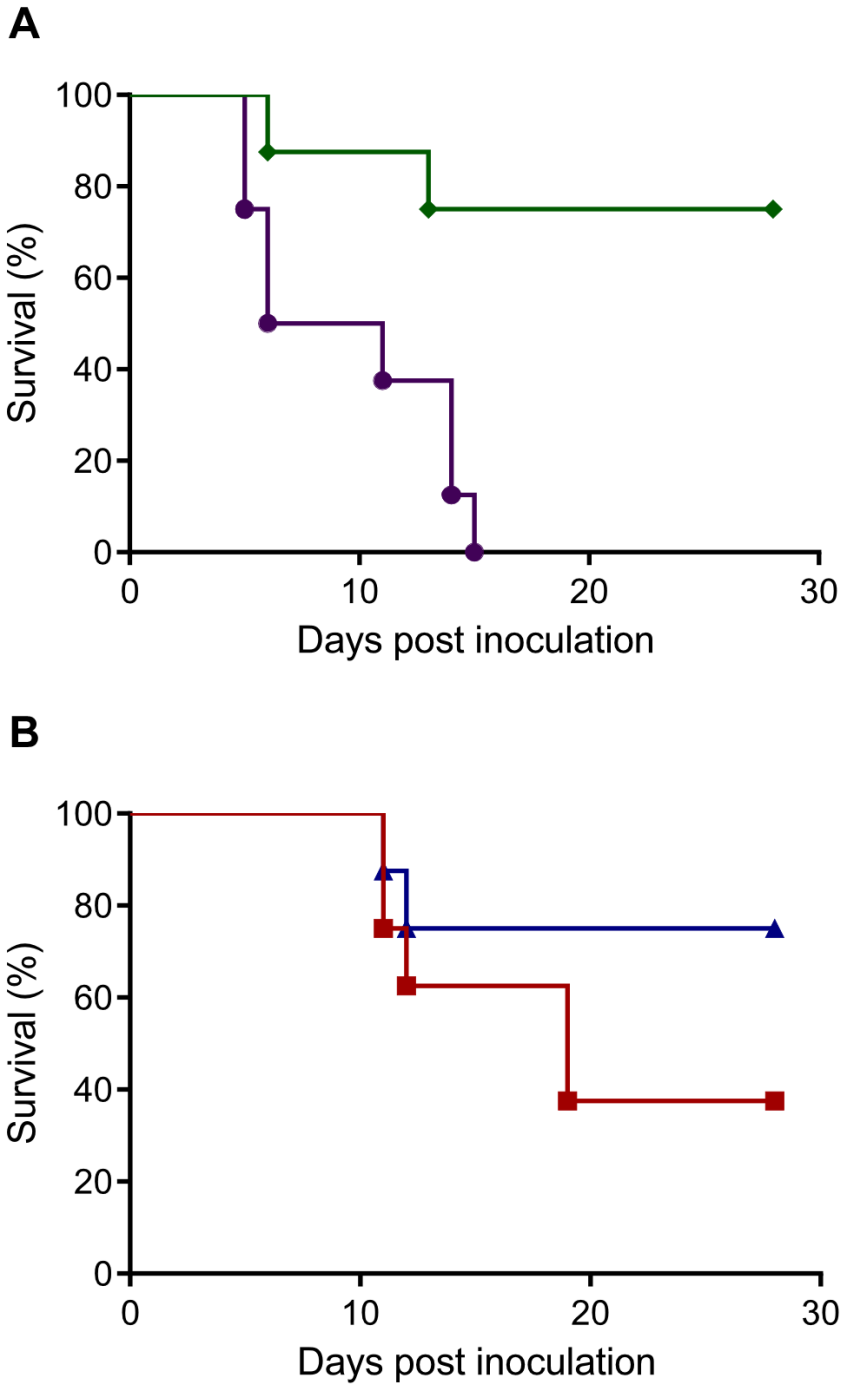
Survival of Syrian hamsters after inoculation with Nipah virus via different routes. Groups of eight hamsters were inoculated with 10^7^ TCID_50_ of Nipah virus (strain Bangladesh/200401066) intranasally (purple line) or esophageally (green line) (A) or via drinking of 10^7^ (blue line) or 5×10^8^ TCID_50_ (red line) of Nipah virus (strain Bangladesh/200401066) (B). The percentage of animals surviving over time is shown.

To assess the ability of Nipah virus to establish an infection upon ingestion of virus, hamsters were inoculated esophageally with 10^7^ TCID_50_ of Nipah virus (strain Bangladesh/200401066). Eight hamsters were monitored daily for signs of disease for up to 28 days post inoculation (dpi). One hamster succumbed to respiratory disease on 6 dpi; a second hamster was euthanized on 12 dpi (Fig. 2A). Two out of the six surviving hamsters seroconverted by 28 dpi, indicating that 4 out of 8 hamsters were likely infected with Nipah virus after esophageal inoculation. For comparison, a group of hamsters was inoculated intranasally with 10^7^ TCID_50_ of Nipah virus. All eight intranasally inoculated animals were euthanized due to severe disease signs between 5 and 14 dpi (Fig. 2A).

**Figure 3:**
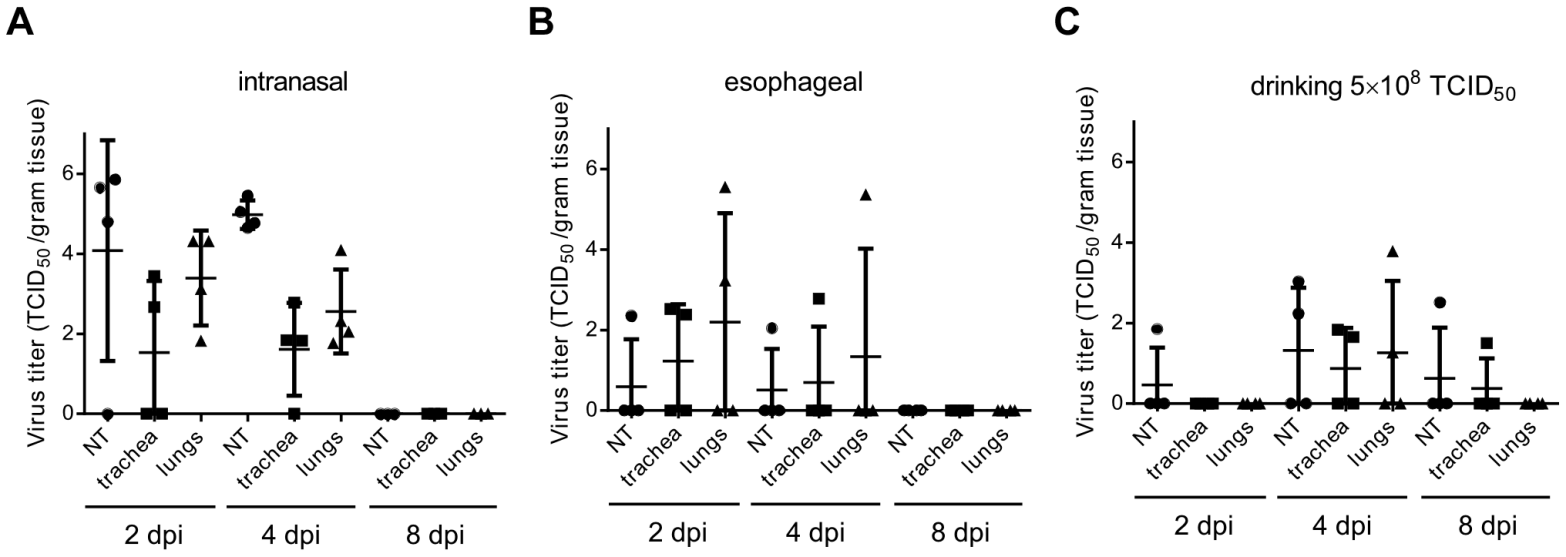
Virus titers in respiratory tissues of hamsters inoculated with Nipah virus. Samples of nasal turbinates (NT; black circles), trachea (black squares) and lungs (black triangles) were collected on 2, 4 and 8 dpi from four animals inoculated intranasally (A) or esophageally (B) with 10^7^ TCID_50_ of Nipah virus (strain Bangladesh/200401066) or with 5×10^8^ TCID_50_ via drinking (C) and virus titers were determined by titration on Vero C1008 cells. Each symbol represents one animal; horizontal line indicates geometric mean; error bars indicate standard deviation.

On 2, 4, and 8 dpi, groups of 4 hamsters inoculated esophageally or intranasally were euthanized and 17 tissues were collected from each hamster for virus titration. In addition, whole blood was collected for analysis of the presence of viral RNA by qRT-PCR. For both inoculation routes, virus was mainly detected in nasal turbinates, trachea and lungs; virus in non-respiratory tissues was observed mostly in animals with evidence of viremia. In the esophageally inoculated hamsters, virus was detected on 2 dpi in 2 out of 4 hamsters (Fig. 3 & Table S1), with viremia detected by qRT-PCR in one of the two remaining hamsters. On 4 dpi, virus was detected in only one hamster, including a low amount of virus in the esophagus of this animal. By 8 dpi, virus could no longer be detected in tissues or blood. On 2 and 4 dpi, virus was detected in respiratory tissues of all intranasally inoculated animals; on 8 dpi, virus could not be detected in the tissues of all three remaining animals, although viremia was detected by RT-PCR in one animal at this time-point (Fig. 3 and Table S1). Histopathological examination did not reveal evidence of virus replication in intestinal tissues in hamsters that were inoculated esophageally (Table S2).

### Nipah virus infection through virus-containing palm sap

Next, the ability of Nipah virus to infect Syrian hamsters via drinking of virus-containing artificial palm sap was assessed. Eight animals were singly housed and their drinking water was replaced with 30 ml artificial palm sap containing 10^7^ TCID_50_ of Nipah virus; animals drank the artificial palm sap containing Nipah virus in approximately 2 days. Animals were assessed for signs of disease and survival for up to 28 days. On 10 and 11 days after supplying the hamsters with artificial palm sap containing Nipah virus, one hamster was euthanized due to neurological signs of disease (Fig. 2B). The remaining six hamsters did not show signs of disease until the end of the experiment at day 28; however, two out of these six hamsters seroconverted, indicating that these animals were likely infected with Nipah virus. Tissues from 4 animals collected on 2 and 4 days after supplying the hamsters with artificial palm sap containing Nipah virus were negative in virus titration and viremia could not be detected by qRT-PCR (Table S1). On day 8, a low amount of virus was detected in the kidney of 1 animal; all other tissues of this hamster and all tissues of three other hamsters were negative (Table S1).

**Figure 4:**
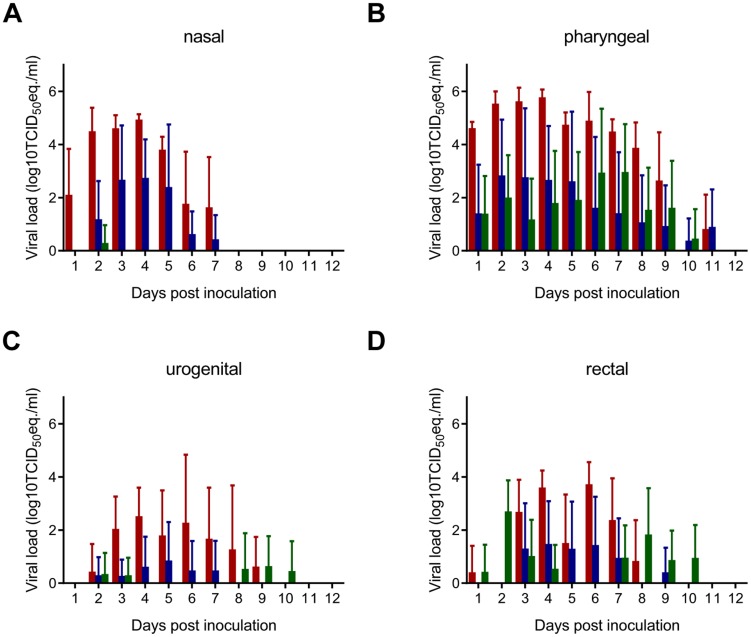
Virus shedding in Syrian hamsters inoculated with Nipah virus via different routes. Groups of eight hamsters were inoculated with 10^7^ TCID_50_ of Nipah virus (strain Bangladesh/200401066) intranasally (red bars) or esophageally (blue bars) and with 5×10^8^ TCID_50_ via drinking (green bars) and nasal (A), pharyngeal (B), urogenital (C) and rectal (D) swabs were collected daily until 12 dpi. Viral load in the swabs was determined as TCID_50_ equivalents by real-time RT-PCR. TCID_50_ equivalents were extrapolated from standard curves generated by adding dilutions of RNA extracted from a Nipah virus stock with a known virus titer in parallel to each run. Geometric mean viral loads are displayed; error bars indicate standard deviation.

Since human-to-human transmission plays an important role in Nipah virus outbreaks in Bangladesh and virus shedding is a prerequisite for transmission, we collected nasal, oropharyngeal, urogenital and rectal swabs daily for nine days after supplying the hamsters with artificial palm sap containing Nipah virus and analyzed these for the presence of Nipah virus RNA. Nipah virus RNA could be detected in only six of the collected swabs; all PCR-positive swabs were oropharyngeal swabs. One of the seroconverted hamsters had a positive swab on 4 and 6 days after supplying it with artificial palm sap; one of the hamsters that was euthanized with neurological signs had positive oropharyngeal swabs on days 5, 6 and 7 after inoculation (data not shown), likely indicating an active infection. In comparison, all intranasally inoculated hamsters shed virus from the nose for up to 7 days and from the throat up to 11 dpi (Fig. 4).

### Increased virus shedding upon exposure to a higher dose of Nipah virus

To determine whether virus shedding increased when animals were supplied with a higher dose of Nipah virus in artificial palm sap, we repeated the drinking experiment with 5×10^8^ TCID_50_ of Nipah virus (strain Bangladesh/200401066) in artificial palm sap. Nasal, oropharyngeal, urogenital and rectal swabs were collected up to 11 days after supplying the hamsters with artificial palm sap. All eight tested hamsters shed virus for several days, mainly via the oropharynx and, at later time points, the intestinal tract (Fig. 4).

**Figure 5:**
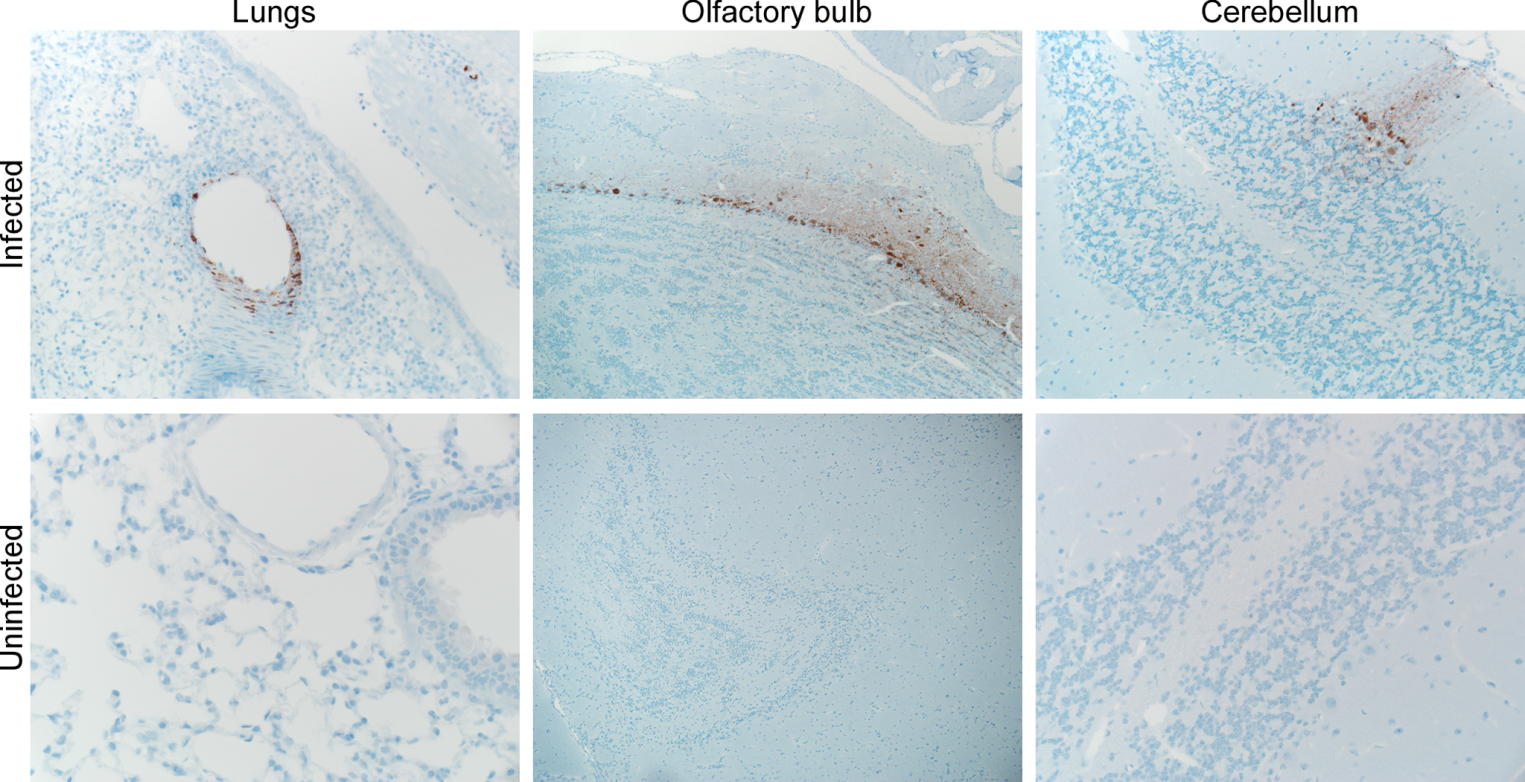
Immunohistochemical analysis of tissues collected from a Syrian hamster inoculated with Nipah virus via drinking. Lungs, olfactory bulb and cerebellum were collected from a hamster inoculated with 5×10^8^ TCID_50_ of Nipah virus via drinking on 8 dpi when the animal was euthanized with neurological signs of diseases. Tissues were stained with a polyclonal antibody against Nipah virus nucleoprotein, which is visible as a red-brown staining.

Between 10 and 18 days after supplying the hamsters with artificial palm sap containing Nipah virus, 5 hamsters had to be euthanized due to the severity of disease; with neurological signs apparent in 4 out of 5 hamsters (Fig. 2B). The three remaining hamsters survived until the end of the experiment at day 28; 2 out of three survivors had seroconverted at that time, indicating that 7 of 8 hamsters likely became infected after drinking artificial palm sap containing a high dose of Nipah virus (strain Bangladesh/200401066). Histopathological examination of tissues collected from hamsters euthanized due to severity of disease revealed signs of bronchointerstitial pneumonia with syncytial cells, fibrin and edema in all 5 hamsters; 2 out of 5 hamsters demonstrated signs of subacute meningitis (Fig. 5 and Table S2).

On day 2 after supplying the hamsters with artificial palm sap containing Nipah virus, virus could only be detected in the nasal turbinates of one out of 4 tested hamsters; all other tested tissues were negative in virus titration and whole blood was negative by RT-PCR (Fig. 3 and Table S1). On day 4, infectious virus was detected in respiratory tissues of 3 out of 4 tested hamsters, but no viral RNA was detected in blood. On day 8, infectious virus was detected in tissues of two out of four hamsters (Fig. 3 and Table S1). Again, histopathological examination of tissues did not implicate involvement of the intestinal tract in virus replication or initiation of infection (Table S2).

### Visualization of deposition of virus in the respiratory tract upon inoculation

**Figure 6:**
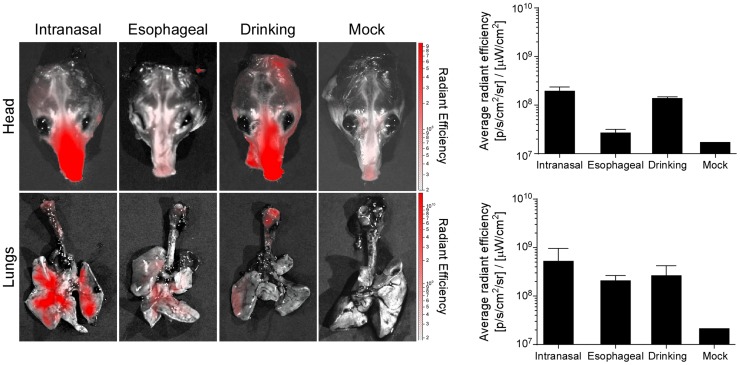
Deposition of virus in the respiratory tract of Syrian hamsters after inoculation with Nipah virus. Nipah virus was purified and fluorescently labeled with Alexa Fluor 680; three hamsters were inoculated intranasally, esophageally or via drinking with 10^7^ TCID_50_ of labeled virus; two hamsters were not inoculated (mock). The head and respiratory tract were collected and imaged in an IVIS Spectrum imager. Scale bar indicates average radiant efficiency. Graph bar indicates average radiant efficiency in the head (top) and lungs (bottom) averaged from 3 animals per inoculation route and 2 animals for mock; error bars indicate standard error of the mean.

To understand how the different inoculation routes could result in virus replication in the lower respiratory tract, we fluorescently labeled Nipah virus and inoculated hamsters with 10^7^ TCID_50_ of this labeled virus intranasally, esophageally or via drinking. After 10 minutes, hamsters were euthanized and the lungs and head prepared for *ex vivo* imaging, to visualize where the inoculum was deposited. In agreement with our tissue distribution data, a large proportion of virus was deposited in the lungs, regardless of whether animals were inoculated intranasally, esophageally or via drinking (Fig. 6). Of note, virus deposition in the stomach could not be assessed due to background fluorescence in this organ. Deposition of virus in the lungs upon esophageal inoculation was likely a result of trace inoculum entering the trachea when the gavage needle was inserted or removed; drinking may result in the generation of aerosols and/or small droplets that were subsequently deposited in the lungs.

### Nipah virus transmission upon drinking of palm sap containing Nipah virus

**Figure 7:**
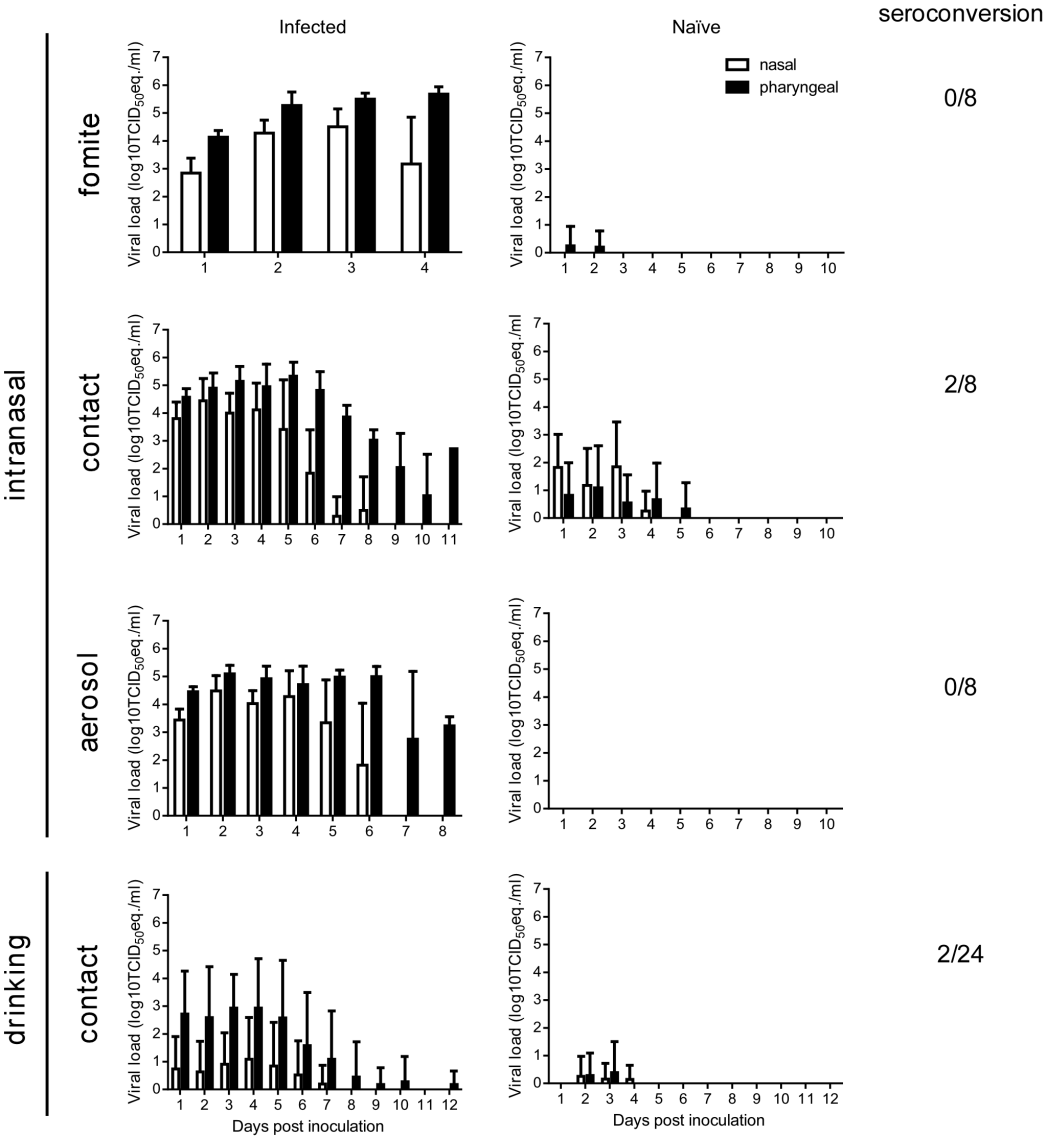
Transmission of Nipah virus (strain Bangladesh/200401066) in Syrian hamsters. Shedding of Nipah virus in inoculated (left panels) and naïve (right panels) animals in fomite, contact and aerosol transmission. Hamsters were inoculated intranasally with 10^7^ TCID_50_ of Nipah virus (strain Bangladesh/200401066) or via drinking of 5×10^8^ TCID_50_ of Nipah virus (strain Bangladesh/200401066) in artificial palm sap; nasal (white bars) and pharyngeal (black bars) swabs were collected daily. Seroconversion in naïve hamsters at 28 dpi as determined by ELISA is indicated on the right as the number of seroconverted hamsters/total number of exposed hamsters. Viral load in the swabs was determined as TCID_50_ equivalents by real-time RT-PCR. TCID_50_ equivalents were extrapolated from standard curves generated by adding dilutions of RNA extracted from a Nipah virus stock with a known virus titer in parallel to each run. Geometric mean viral loads are displayed; error bars indicate standard deviation.

We have recently shown that Nipah virus (strain Malaysia) is transmitted between Syrian hamsters primarily through direct contact [14]. We have also determined the transmission route of Nipah virus (strain Bangladesh/200401066). Groups of eight hamsters were inoculated intranasally with 10^7^ TCID_50_ of Nipah virus (strain Bangladesh/200401066) and singly housed to examine transmission via fomites, direct contact or aerosols as described previously [14]. At 1 dpi, a naïve hamster was added to each cage. Inoculated and naïve hamsters were swabbed daily. At 28 dpi all naïve hamsters were euthanized and sera were tested for antibodies to Nipah virus. None of the hamsters exposed through fomites or aerosols seroconverted (Fig. 7). Two out of 8 hamsters exposed via direct contact seroconverted (Fig. 7), indicating that transmission of Nipah virus (strain Malaysia) and Nipah virus (strain Bangladesh/200401066) occurs via a similar route and at a similar rate [14].

Next, we set out to determine if hamsters infected with Nipah virus through drinking of artificial palm sap containing Nipah virus could transmit the virus to naïve hamsters via direct contact. Of note, a larger number of animals was used in this experiment since the hamsters infected with Nipah virus through drinking of artificial palm sap shed a lower amount of virus than intranasally inoculated hamsters (Fig. 4) and transmission was therefore expected to be less efficient. Twenty-four hamsters were supplied with 30 ml of artificial palm sap containing 5×10^8^ TCID_50_ of Nipah virus (strain Bangladesh/200401066). After two days, when the hamsters had drunk the artificial palm sap, drinking bottles were replaced with bottles containing water and a naïve hamster was added to each cage. At 28 dpi the naïve hamsters were euthanized and sera were collected. Out of 24 naïve hamsters, 2 hamsters showed presence of antibodies directed against Nipah virus in ELISA, likely indicating that Nipah virus was transmitted to these hamsters.

## Discussion

Epidemiological investigations in Bangladesh suggest that Nipah virus is introduced into the human population via the consumption of raw date palm sap. This study provides the first experimental evidence for the transmission of Nipah virus via the consumption of palm sap containing Nipah virus, resulting in neurological signs of disease in Syrian hamsters. Although these findings do not directly demonstrate that date palm sap contaminated with Nipah virus by bats is the origin of Nipah virus outbreaks in Bangladesh, they provide experimental support for the current hypothesis, based on epidemiological observations, of the zoonotic introduction of Nipah virus via contaminated date palm sap.

Nipah virus was very stable in artificial palm sap, likely due to its neutral pH and high sugar content. Nipah virus was preserved much better in artificial palm sap than on the surface of fruit or in fruit juice [18]. In fruit bats, Nipah virus is predominantly shed via urine [11], [19], [20]. Although bat urine itself may not preserve Nipah virus very well [18], the urine would be quickly diluted in the palm sap. Thus, palm sap is likely a very suitable carrier for foodborne transmission of Nipah virus. The rapid decrease in virus titer upon heat treatment of Nipah virus-containing palm sap suggests that this might reduce the risk of Nipah virus transmission to humans.

Based on virus distribution in tissues of infected hamsters and the presence of vRNA mainly in throat swabs rather than urogenital or rectal swabs, the porte d'entrée for the initial Nipah virus infection upon drinking of artificial palm sap containing Nipah virus was the respiratory tract rather than the intestinal tract. This finding was further strengthened using fluorescently labeled Nipah virus to visualize the deposition of virus upon inoculation via the nose, gavage or upon drinking. These experiments clearly showed that during drinking the virus does not only end up in the intestinal tract but some of the volume is also deposited in the lungs, thereby explaining the replication of virus in respiratory tissues. However, the main disease manifestation in hamsters infected through drinking was the development of neurological signs, suggesting that the animals became infected with a relatively low dose, despite the high amount of virus present in the artificial palm sap. Previous studies have shown that inoculation of hamsters with a low dose of Nipah virus results in neurological signs of disease, whereas a high dose results in respiratory disease [15], [21].

In one of the hamsters infected with Nipah virus through drinking of palm sap, virus was detected by immunohistochemistry in the olfactory bulb, indicating that virus traveled from olfactory neurons in the nasal turbinates through the cribriform plate into the olfactory bulb and from there further into the central nervous system. Although this route of Nipah virus into the brain has been described before [22], [23], this is the first time it is described in animals without deliberate inoculation of the nasal cavity.

Besides the ability of Nipah virus to infect hamsters via the drinking route, we showed here that Syrian hamsters infected with Nipah virus through drinking of palm sap containing Nipah virus can transmit the virus through direct contact with naïve hamsters. Transmission upon drinking of Nipah virus-containing artificial palm sap was less efficient than upon intranasal inoculation with Nipah (strain Bangladesh/200401066), likely as a result of decreased virus shedding upon infection through drinking. Within the transmission model for Nipah virus in Syrian hamsters no differences were observed in transmission route or efficiency between the Malaysian [14] and a virus isolate from Bangladesh. Although experimental infection of ferrets suggested that there is increased oral shedding with a Nipah virus isolate from Bangladesh as compared to a virus isolate from Malaysia, this study did not include transmission experiments [24]. Thus it is currently not clear whether the differences in virus shedding observed in the ferret model result in differences in transmission efficiency. Different Nipah virus isolates from several Nipah virus outbreaks in Bangladesh would have to be tested in the different animal models to assess the transmission efficiency of this virus properly.

Prophylactic or therapeutic intervention measures are currently not available to prevent, treat or contain zoonotic transmission of Nipah virus. Moreover, medical interventions might be difficult to implement in rural outbreak areas. Therefore, our best hope to prevent or intervene in future outbreaks of Nipah virus lies in the potential to efficiently block zoonotic and human-to-human transmission and thereby spread of the outbreak. Currently, efforts are underway in Bangladesh to prevent zoonotic transmission of Nipah virus from fruit bats to people by restricting access of bats to date palm collection pots and thereby preventing contamination of the date palm sap with Nipah virus [8], [25]. The data presented here stress the importance of these efforts in Bangladesh in the prevention of Nipah virus outbreaks.

## Materials and Methods

### Ethics statement

All animal experiments were approved by the Institutional Animal Care and Use Committee of the Rocky Mountain Laboratories, and performed following the guidelines of the Association for Assessment and Accreditation of Laboratory Animal Care, International (AAALAC) by certified staff in an AAALAC-approved facility.

### Virus and cells

Nipah virus (strain Bangladesh/200401066) was kindly provided by the Special Pathogens Branch of the Centers for Disease Control and Prevention, Atlanta, Georgia, United States. This strain (SPBLOG# 200401066) was isolated from a throat swab collected from patient #3001 on January 22 2004 in Bangladesh. This patient was a 10-year old male who developed altered mental status on January 21 and cough and breathing difficulties later that day. The patient was admitted to Goalando Hospital, Bangladesh, on January 22. None of the patient's contacts developed Nipah virus infection; the patient is presumed to have been infected via direct spillover from the bat reservoir (dr. Steve Luby, personal communication). The virus isolate was propagated in Vero C1008 cells in DMEM (Sigma) supplemented with 10% fetal calf serum, 1 mM L-glutamine (Lonza), 50 U/ml penicillin and 50 µg/ml streptomycin (Gibco). For fluorescent labelling of virus, Nipah virus-containing cell supernatant was cleared by low speed centrifugation and virus was pelleted by spinning 2 hours at 21000 rpm in the ultracentrifuge. The pellet was resuspended in 1 ml PBS and loaded onto a 20%–60% (w/w) sucrose gradient and centrifuged overnight at 39000 rpm. The virus fraction was collected and pelleted once again by centrifuging 2 hours at 21000 rpm; the pellet was resuspended in 1 ml PBS. Purified Nipah virus particles were labeled using an Alexa Fluor 680 Protein Labeling Kit (Molecular Probes). Excess dye was removed by dialyzing against PBS.

### Artificial palm sap

Artificial palm sap was prepared based on a literature report [17] and consisted of 13% sucrose (w/v) and 0.21% BSA in water. The pH of the artificial palm sap was 7 without any adjustments.

### Stability of Nipah virus in artificial palm sap

Nipah virus (strain Bangladesh/200401066) was added to artificial palm sap at the desired concentration, aliquotted into 1 ml aliquots and incubated at 22°C or 28°C for up to eight days. The stability data were analyzed using the linear regression model in the GraphPad prism 6 software package. For inactivation of Nipah virus in artificial palm sap, Nipah virus (strain Bangladesh/200401066) was added to artificial palm sap at 10^7^ TCID_50_/ml, aliquotted into 1 ml aliquots and incubated at 70°C or 100°C for up to one hour.

### Animal experiment: Different inoculation routes

Four groups of 40 6–8 week old female Syrian hamsters (HsdHan^tm^:AURA, Harlan Laboratories) were inoculated with Nipah virus via different routes. One group received 10^7^ TCID_50_ Nipah virus (strain Bangladesh/200401066) via intranasal inoculation in a total volume of 80 µl. One group received 10^7^ TCID_50_ Nipah virus (strain Bangladesh) via gavage in a total volume of 500 µl. The remaining two groups received 10^7^ and 5×10^8^ TCID_50_ Nipah virus (strain Bangladesh/200401066), respectively through drinking of artificial palm sap. Animals were housed singly and supplied with 30 ml artificial palm sap containing a total dose of 10^7^ or 5×10^8^ TCID_50_ Nipah virus instead of drinking water. When animals had drunk all artificial palm sap, in about 2 days, they were again supplied with drinking water. Nasal, oral, urogenital and rectal swabs were collected daily from eight hamsters inoculated via all four different routes. Swabs were collected in vials containing 1 ml DMEM supplemented with 50 U/ml penicillin and 50 µg/ml streptomycin. On days 2, 4, 8, 12 and 28 post inoculation 8 animals from each inoculation group were euthanized and blood, trachea, lungs, heart, liver, spleen, kidney, esophagus, stomach, duodenum, jejunum, ileum, cecum, colon (proximal and distal), bladder, brain and nasal turbinates were collected for virological (4 animals/time point) and histopathological (4 animals/time point) analysis. Hamsters used for histopathological analysis were anaesthetized using ketamine (80–100 mg/kg) and xylazine (7–10 mg/kg) and perfused with PBS containing 5 mM EDTA, followed by 4% paraformaldehyde. Tissues of interest were then further fixed according to BSL4 standard operating procedures for a minimum of 7 days in 10% neutral buffered formalin.

### Animal experiment: *Ex vivo* imaging

To visualize the deposition of virus in the hamster respiratory tract after inoculation, 3 hamsters per inoculation route were inoculated intranasally, esophageally and via drinking as described above with 10^7^ TCID_50_ of fluorescently labeled Nipah virus (strain Bangladesh/200401066). To prevent fusion of virus particles with target cells, hamsters were euthanized ten minutes after inoculation and the respiratory tract was excised; *ex vivo* imaging was subsequently performed on the head and respiratory tract in an IVIS Spectrum imager (PerkinElmer). Images were acquired using an excitation wavelength of 675 nm with an emission scan at 720, 740, 760 and 780 nm at field of view B (6.6 cm) in auto-exposure mode with medium binning, f-stop 3. Following acquisition, images were unmixed in Living Image 4.2 with tissue autofluorescence subtracted. The resulting AF680 image was used for subsequent analysis. Rectangular Regions of Interest were drawn around the entire lung, trachea or nasal tract. The resulting average radiant efficiency was used to determine the quantity of labelled virus that was detected in the respiratory tract (combined trachea and lungs) or in the nasal tract (head).

### Animal experiment: Transmission route

To determine the transmission route for Nipah virus (strain Bangladesh/200401066) we used the recently described Syrian hamster transmission model [14]. For fomite transmission experiments, eight 6–8 week old female singly housed Syrian hamsters, housed in a plastic cage with wood shavings, a feeder and a water bottle, were inoculated intranasally with 10^7^ TCID_50_ of Nipah virus (strain Bangladesh) in a total volume of 80 µl. On day 4 post inoculation, hamsters were euthanized and a single naïve hamster was placed in each cage. For direct contact transmission experiments, eight 6–8 week old female singly housed Syrian hamsters were inoculated intranasally with 10^7^ TCID_50_ of Nipah virus (strain Bangladesh/200401066) in a total volume of 80 µl. On day 1 post inoculation, a naïve hamster was added to each cage. For aerosol transmission experiments, eight 6–8 week old female Syrian hamsters were inoculated intranasally with 10^7^ TCID_50_ of Nipah virus (strain Bangladesh/200401066) in a total volume of 80 µl and singly housed in specially designed aerosol transmission cages. On 1 dpi, a naïve hamster was placed on the opposite side of the inoculated hamster. The hamsters were separated by two stainless steel grids, allowing airflow from the inoculated to the naive hamster but preventing direct contact and fomite transmission. In all transmission experiments, nasal and oropharyngeal swabs were obtained from inoculated and naïve hamsters daily and the bodyweight of naïve hamsters was determined. Upon signs of severe disease, inoculated and naïve hamsters were euthanized; remaining hamsters were euthanized four weeks post exposure.

### Animal experiment: Transmission after inoculation via drinking

To determine whether Nipah virus (strain Bangladesh/200401066) is transmitted via direct contact after infection through palm sap containing Nipah virus, 24 6–8 week old female singly housed Syrian hamsters were supplied with 30 ml artificial palm sap containing 5×10^8^ TCID_50_ of Nipah virus (strain Bangladesh/200401066) instead of drinking water. When animals had drunk all artificial palm sap they were again supplied with drinking water and a naïve hamster was added to each cage. Nasal and oropharyngeal swabs were obtained from inoculated and naïve hamsters daily and bodyweight of naïve hamsters was determined. On signs of severe disease, inoculated and naïve hamsters were euthanized; remaining hamsters were euthanized four weeks post exposure.

### Virus titrations

Virus titrations were performed by end-point titration in VeroC1008 cells. VeroC1008 cells were inoculated with tenfold serial dilutions of swab medium or tissue homogenates. One hour after inoculation, the inoculum was removed and replaced with 200 µl DMEM supplemented with 10% fetal calf serum, 1 mM L-glutamine (Lonza), 50 U/ml penicillin and 50 µg/ml streptomycin (Gibco). Five days after inoculation, cytopathic effect (CPE) was scored and the TCID_50_ was calculated from 5 replicates by the method of Spearman-Karber. Tissue homogenates were prepared by adding 1 ml DMEM to the weighed tissue and homogenizing using a TissueLyzer II (Qiagen). Homogenates were centrifuged to clear the homogenate before inoculating cells.

### Histopathology and immunohistochemistry

Histopathology and immunohistochemistry was performed on hamster tissues. Necropsies and tissue sampling were performed according to a standard protocol approved by the Institutional Biosafety Committee. After fixation for 7 days in 10% neutral-buffered formalin and embedding in paraffin, tissue sections were stained with hematoxylin and eosin (H&E) and an immunohistochemical method using a rabbit polyclonal antiserum against the Nipah virus nucleoprotein [26] (1∶5000; kindly provided by L. Wang, CSIRO Livestock Industries, Australian Animal Health Laboratory, Australia) as a primary antibody for detection of Nipah virus antigen. For the histopathological analysis of the nasal turbinates (NT) whole hamster skulls were used. The skulls were decalcified using a 20% EDTA solution in sucrose (Newcomer Supply) and allowed to sit at room temperature for 3 weeks. The 20% EDTA/sucrose solution was changed 2 times prior to gross sectioning the skull. The following tissues were examined: NT, trachea and lungs. Lesions were assigned a subjective score from 0 to 4 based on the percentage of the tissue that was immunopositive. The slides were evaluated by a board-certified veterinary pathologist.

### Quantitative PCR

RNA was extracted from swab samples using the NucleoSpin 96 Virus Core kit (Macherey-Nagel) and a Corbett Robotics model CAS 1820 automatic RNA extractor. RNA was eluted in 100 µl. 5 µl RNA was used in a one-step real-time RT-PCR targeted at the NP gene using the Rotor-Gene probe kit (Qiagen) according to instructions of the manufacturer (primer and probe sequences are available on request). In each run, standard dilutions of a titered virus stock were run in parallel, to calculate TCID_50_ equivalents in the samples.

### ELISA

Antibody responses were measured in an enzyme-linked immunosorbent assay (ELISA) using inactivated Nipah virus (strain Malaysia) as the antigen. Nipah virus-containing cell culture supernatant was concentrated and purified by centrifuging for two hours at 21000 rpm over a 20% sucrose cushion. The pellet was resuspended in PBS and Triton X-100 was added to a final concentration of 1%; the preparation was then inactivated with γ-radiation according to standard operating procedures. This suspension was used to coat immuno 96 microwell maxisorp plates (NUNC) at 4°C overnight. Subsequently, plates were blocked with 5% skim milk in PBS containing 0.05% Tween 20 (PBST) for 1.5 hours at 4°C. After 3 washes with PBST, 50 µL of diluted serum samples were added, and the plates were incubated for 1 hour at 37°C. Bound antibodies were detected after 3 washes using an anti-hamster secondary antibody conjugated with horseradish peroxidase (HRP; KPL). Following incubation for 1 hour at 37°C, bound HRP was detected using the ABTS Peroxidase Substrate System (KPL). The absorbance at 405 nm was measured using a microplate spectrophotometer. Sera were considered positive when absorbance was higher than three standard deviations above the mean of negative control sera.

## Supporting Information

Figure S1 Stability of Nipah virus in artificial palm sap. (A) Nipah virus (strain Bangladesh/200401066) was diluted to 10^7^ (black triangles) and 10^5^ (black squares) TCID_50_/ml in artificial palm sap and left at 28°C for 8 days. Samples were taken at the indicated time points and virus titer in those samples was determined by titration on Vero C1008 cells. The stability data were analyzed using the linear regression model in the GraphPad prism 6 software package. The regression line (solid) is plotted together with the 95% confidence interval (dotted line). (B) Nipah virus (strain Bangladesh/200401066) was diluted to 10^7^ TCID_50_/ml in artificial palm sap and left at 70°C (green line) or 100°C (blue line) for 1 hour. Samples were taken at the indicated time points and virus titer in those samples was determined by titration on Vero C1008 cells. Geometric mean titers were calculated from three replicates; error bars indicate standard deviation; dotted line indicates the cut off value of the assay.
(TIF)

Table S1 Tissue distribution of Nipah virus in hamsters inoculated intranasally, esophageally or via drinking as determined by virus titration. Numbers in the table indicate number of animals in which virus was detected in the indicated tissues at the indicated time points; total number of animals is indicated at the top of the column. ^1^ Viremia was determined by real-time RT-PCR.
(DOCX)

Table S2 Tissue distribution of Nipah virus in hamsters inoculated intranasally, esophageally or via drinking as determined by immunohistochemistry. Numbers in the table indicate number of animals in which viral antigen was detected in the indicated tissues at the indicated time points; total number of animals is indicated at the top of the column. ^1^ Tissues were collected when animals were euthanized due to severity of disease signs. ^2^ nc: not collected.
(DOCX)
