# Supplementary material for: Foodborne Transmission of Nipah Virus in Syrian Hamsters
Source: PLoS Pathog. 2014 Mar 13;10(3):e1004001. doi: 10.1371/journal.ppat.1004001 (PMC3953481; doi:10.1371/journal.ppat.1004001)
Supplement: Table S1 — Tissue distribution of Nipah virus in hamsters inoculated intranasally, esophageally or via drinking as determined by virus titration. Numbers in the table indicate number of animals in which virus was detected in the indicated tissues at the indicated time points; total number of animals is indicated at the top of the column. 1 Viremia was determined by real-time RT-PCR. (DOCX) [file ppat.1004001.s002.docx]

**Table S1.** Tissue distribution of Nipah virus in hamsters inoculated intranasally, esophageally or via drinking as determined by virus titration. Numbers in the table indicate number of animals in which virus was detected in the indicated tissues at the indicated time points; total number of animals is indicated at the top of the column.

|  | Intranasal | | | Esophageal | | | Drinking  (10^7^ TCID_50_) | | | Drinking  (5x10^8^ TCID_50_) | | |
| --- | --- | --- | --- | --- | --- | --- | --- | --- | --- | --- | --- | --- |
|  | 2 dpi | 4 dpi | 8 dpi | 2 dpi | 4 dpi | 8 dpi | 2 dpi | 4 dpi | 8 dpi | 2 dpi | 4 dpi | 8 dpi |
|  | (n=4) | (n=4) | (n=3) | (n=4) | (n=4) | (n=4) | (n=4) | (n=4) | (n=4) | (n=4) | (n=4) | (n=3) |
| Trachea | 2 | 3 | 0 | 2 | 1 | 0 | 0 | 0 | 0 | 0 | 2 | 1 |
| Lung | 4 | 4 | 0 | 2 | 1 | 0 | 0 | 0 | 0 | 0 | 2 | 0 |
| Heart | 1 | 0 | 0 | 1 | 1 | 0 | 0 | 0 | 0 | 0 | 0 | 0 |
| Liver | 1 | 0 | 0 | 0 | 0 | 0 | 0 | 0 | 0 | 0 | 0 | 0 |
| Spleen | 0 | 0 | 0 | 0 | 1 | 0 | 0 | 0 | 0 | 0 | 0 | 0 |
| Kidney | 0 | 0 | 0 | 0 | 0 | 0 | 0 | 0 | 1 | 0 | 0 | 1 |
| Esophagus | 0 | 0 | 0 | 0 | 1 | 0 | 0 | 0 | 0 | 0 | 0 | 0 |
| Stomach | 0 | 0 | 0 | 0 | 0 | 0 | 0 | 0 | 0 | 0 | 0 | 0 |
| Duodenum | 0 | 0 | 0 | 0 | 0 | 0 | 0 | 0 | 0 | 0 | 0 | 0 |
| Jejunum | 0 | 0 | 0 | 0 | 0 | 0 | 0 | 0 | 0 | 0 | 0 | 0 |
| Ileum | 0 | 0 | 0 | 0 | 0 | 0 | 0 | 0 | 0 | 0 | 0 | 0 |
| Cecum | 0 | 0 | 0 | 0 | 0 | 0 | 0 | 0 | 0 | 0 | 0 | 0 |
| Colon (ascending) | 0 | 0 | 0 | 0 | 0 | 0 | 0 | 0 | 0 | 0 | 0 | 0 |
| Colon (descending) | 0 | 0 | 0 | 0 | 0 | 0 | 0 | 0 | 0 | 0 | 0 | 0 |
| Bladder | 0 | 0 | 0 | 0 | 1 | 0 | 0 | 0 | 0 | 0 | 0 | 0 |
| Brain | 1 | 0 | 0 | 0 | 0 | 0 | 0 | 0 | 0 | 0 | 0 | 0 |
| Nasal turbinates | 3 | 4 | 0 | 1 | 1 | 0 | 0 | 0 | 0 | 1 | 2 | 1 |
| Viremia^1^ | 1 | 0 | 1 | 1 | 1 | 0 | 0 | 0 | 0 | 0 | 0 | 0 |

^1^ Viremia was determined by real-time RT-PCR
