# Supplementary material for: Foodborne Transmission of Nipah Virus in Syrian Hamsters
Source: PLoS Pathog. 2014 Mar 13;10(3):e1004001. doi: 10.1371/journal.ppat.1004001 (PMC3953481; doi:10.1371/journal.ppat.1004001)
Supplement: Table S2 — Tissue distribution of Nipah virus in hamsters inoculated intranasally, esophageally or via drinking as determined by immunohistochemistry. Numbers in the table indicate number of animals in which viral antigen was detected in the indicated tissues at the indicated time points; total number of animals is indicated at the top of the column. 1 Tissues were collected when animals were euthanized due to severity of disease signs. 2 nc: not collected. (DOCX) [file ppat.1004001.s003.docx]

**Table S2.** Tissue distribution of Nipah virus in hamsters inoculated intranasally, esophageally or via drinking as determined by immunohistochemistry. Numbers in the table indicate number of animals in which viral antigen was detected in the indicated tissues at the indicated time points; total number of animals is indicated at the top of the column.

|  | Intranasal | | Esophageal | | | Drinking (10^7^ TCID_50_) | | | Drinking (5x10^8^ TCID_50_) | | | |
| --- | --- | --- | --- | --- | --- | --- | --- | --- | --- | --- | --- | --- |
|  | 2 dpi | 4 dpi | 2 dpi | 4 dpi | 8 dpi | 2 dpi | 4 dpi | 8 dpi | 2 dpi | 4 dpi | 8 dpi | euth.^1^ |
|  | (n=4) | (n=4) | (n=4) | (n=4) | (n=4) | (n=4) | (n=4) | (n=4) | (n=4) | (n=4) | (n=3) | (n=5) |
| Trachea | 1 | 2 | 0 | 0 | 0 | 0 | 0 | 0 | 0 | 0 | 0 | nc^2^ |
| Lung | 4 | 4 | 3 | 1 | 0 | 0 | 0 | 2 | 0 | 0 | 1 | 1 |
| Liver | 0 | 1 | 0 | 0 | 0 | 0 | 0 | 1 | 0 | 0 | 0 | nc |
| Spleen | 0 | 1 | 0 | 0 | 0 | 0 | 0 | 1 | 0 | 0 | 0 | nc |
| Kidney | 0 | 3 | 0 | 0 | 0 | 0 | 0 | 2 | 0 | 0 | 0 | nc |
| Esophagus | 0 | 0 | 0 | 0 | 0 | 0 | 0 | 0 | 0 | 0 | 0 | nc |
| Stomach | 0 | 0 | 0 | 0 | 0 | 0 | 0 | 1 | 0 | 0 | 0 | nc |
| Duodenum | 0 | 0 | 0 | 0 | 0 | 0 | 0 | 0 | 0 | 0 | 0 | nc |
| Jejunum | 0 | 1 | 0 | 0 | 0 | 0 | 1 | 0 | 0 | 0 | 0 | nc |
| Ileum | 0 | 0 | 0 | 0 | 0 | 0 | 1 | 0 | 0 | 0 | 0 | nc |
| Cecum | 0 | 0 | 0 | 0 | 0 | 0 | 0 | 0 | 0 | 0 | 0 | nc |
| Colon (ascending) | 0 | 1 | 0 | 0 | 0 | 0 | 0 | 0 | 0 | 0 | 0 | nc |
| Colon (descending) | 0 | 0 | 0 | 0 | 0 | 0 | 0 | 0 | 0 | 0 | 0 | nc |
| Bladder | 0 | 0 | 0 | 0 | 0 | 0 | 0 | 0 | 0 | 0 | 0 | nc |
| Brain | 0 | 0 | 0 | 0 | 0 | 0 | 0 | 0 | 0 | 0 | 0 | 4 |
| Nasal turbinates | 4 | 4 | 1 | 1 | 0 | 0 | 0 | 0 | 0 | 0 | 0 | 0 |

^1^ Tissues were collected when animals were euthanized due to severity of disease signs.

^2^ nc: not collected
